# Intranasal Immunization with DnaK Protein Induces Protective Mucosal Immunity against Tuberculosis in CD4-Depleted Mice

**DOI:** 10.3389/fcimb.2018.00031

**Published:** 2018-02-08

**Authors:** Yu-Min Chuang, Michael L. Pinn, Petros C. Karakousis, Chien-Fu Hung

**Affiliations:** ^1^Department of Pathology, Johns Hopkins University School of Medicine, Baltimore, MD, United States; ^2^Department of Medicine, Johns Hopkins University School of Medicine, Baltimore, MD, United States; ^3^Department of International Health, Johns Hopkins Bloomberg School of Public Health, Baltimore, MD, United States

**Keywords:** tuberculosis, intranasal, DnaK, vaccine, immunodeficiency

## Abstract

*Mycobacterium tuberculosis* (Mtb) remains a global health challenge due to the limited efficacy of the Mtb vaccine in current use, Bacillus Calmette–Guérin (BCG). To date, there is no available vaccine for immunocompromised individuals. Thus, there is an urgent need to develop a new vaccine candidate which can induce mucosal immunity in hosts with different immune statuses. DnaK (HSP70) has been shown to induce protective immunity against Mtb infection when administered by DNA vaccine; however, the protection is inferior to that induced by the BCG vaccine. In our study, we vaccinated C57BL/6J mice with DnaK protein alone. Subcutaneous or intranasal vaccination with DnaK generated IFNγ-secreting CD4^+^ T cells in the spleen, but only intranasal vaccination generated IL-17-releasing CD4^+^ T cells in the lungs, even when circulating CD4^+^ T cells were diminished. Furthermore, intranasal vaccination with DnaK generated tissue resident CD4^+^ T cells in the lungs. Vaccination with DnaK alone resulted in protective immunity comparable to BCG vaccination against tuberculosis in mice. Our results demonstrate that intranasal vaccination with DnaK can generate mucosal immunity in immunocompromised or immunocompetent mice and DnaK vaccination can generate protection against Mtb similar to BCG, underscoring its potential utility as an Mtb vaccine candidate in humans.

## Introduction

Tuberculosis (TB) is one of the primary causes of morbidity and mortality due to infectious diseases worldwide. Despite substantial research in the field, the *Mycobacterium bovis* Bacillus Calmette–Guérin (BCG) vaccine remains the only licensed preventive vaccine against TB, which was introduced into clinical practice nearly a century ago. Although the BCG vaccine may protect children against some forms of TB, including central nervous system infections, its protective efficacy is highly variable and the vaccine does not confer protection against pulmonary TB in adults (Andersen and Doherty, 2005). The suboptimal efficacy of the BCG vaccine has been attributed to previous exposure to cross-reacting non-tuberculosis mycobacteria (Andersen and Doherty, 2005), chronic helminth infections (Elias et al., 2008), and the absence of key *Mycobacterium tuberculosis* (Mtb)-related antigens in the vaccine strain (Kalra et al., 2007). In addition, the live attenuated strain *M. bovis* BCG can escape host immunity and interfere with antigen presentation, contributing to reduced activation of T cells (Sendide et al., 2005; Russell et al., 2007; Pecora et al., 2009). The protective efficacy of the BCG vaccine is dependent on the induction of T-helper 1 (Th1) immune responses (Black et al., 2002), and recent studies suggest that boosting pre-exposure Th17 responses further enhances its protective efficacy (Chatterjee et al., 2011; Kleinnijenhuis et al., 2014). Multiple strategies have been used to improve the efficacy of the BCG vaccine, including genetic modification of the BCG strain (Bottai et al., 2015; Saiga et al., 2015), prime-boost vaccination with a modified vaccinia virus Ankara (MVA) (Fletcher et al., 2016), and immunomodulatory techniques to enhance BCG immunogenicity (Jagannath et al., 2009; Speth et al., 2016). For example, toll-like receptor (TLR) agonists, which can activate innate immunity and promote antigen presentation (Lahiri et al., 2008), have shown promise as adjuvants in preclinical studies, although their immunological effects are variable (Gupta et al., 2016), and they may induce toxicity in humans (Steinhagen et al., 2011).

One possible explanation for the relatively modest protective efficacy of the BCG vaccine is that it is commonly administered via the subcutaneous route, which fails to induce significant mucosal immunity (Aguilo et al., 2014; Perdomo et al., 2016). On the other hand, mucosal vaccination with BCG generates IL-17-secreting antigen-specific CD4^+^ T cells in the mucosa and offers superior protection to challenge with virulent Mtb as compared to subcutaneous BCG vaccination (Aguilo et al., 2016; Perdomo et al., 2016). However, even if the efficacy of the BCG vaccine could be improved by use of alternative routes of administration, there are remaining concerns about the safety of using a live attenuated strain, since BCG vaccination has been associated with significant mortality and morbidity, particularly in children with primary immune deficiencies (Marciano et al., 2014) and those with AIDS (Hesseling et al., 2007), which are among the populations most susceptible to TB. Therefore, there is an urgent need to develop novel TB vaccination strategies to enhance vaccine potency among vulnerable immune-competent and immune-deficient populations.

Mycobacterial heat shock proteins serve as molecular chaperones for other proteins during stress conditions and help to recycle damaged proteins (Kim et al., 2013; Balchin et al., 2016). DnaK (HSP70) is a member of a small chaperone family, which is conserved in both prokaryotes and eukaryotes (Kim et al., 2013; Balchin et al., 2016). In Mtb, DnaK is encoded by an essential gene (Sassetti et al., 2003; Griffin et al., 2011), which is required for bacterial growth and proper protein folding (Fay and Glickman, 2014). DnaK can also activate innate immunity through TLR-2 and TLR-4 (Bulut et al., 2005; Tsan and Gao, 2009). Vaccination with DnaK linked innate and adaptive immunity and had an adjuvant effect during vaccination (Suzue and Young, 1996; Bulut et al., 2005). HSP70 fused with human papillomavirus (HPV) E6 as a DNA vaccine was found to generate HPV-specific T-cell responses in patients (Trimble et al., 2009), and mycobacterial HSP70 has been shown to induce protective immunity by DNA vaccination in mice (Tascon et al., 1996; Lowrie et al., 1997). Furthermore, a DNA vaccine encoding HSP70, HSP65 and Apa given as a booster vaccine after BCG vaccination showed enhanced protection in the murine TB model compared to BCG vaccination alone (Ferraz et al., 2004). However, the protective effect was variable and inferior to BCG when vaccination with single antigens was administered (Lowrie et al., 1997). In the current study, we demonstrated that vaccination with recombinant DnaK protein alone by the subcutaneous (SC) or intranasal (IN) route can confer protective immunity. Only intranasal vaccination with DnaK could generate mucosal-associated IL-17-secreting CD4^+^ T cells, even in systemic CD4^+^-depleted mice, offering a potential new vaccine strategy against TB.

## Materials and methods

### DnaK protein preparation

H37Rv DnaK cloned into pMCSG73 (DNASU, clone: MtCD00587183) was used for expression of recombinant DnaK protein. The resulting plasmids were used to transform *E. coli BL21* (DE3) RP competent cells (Stratagene). The transformed bacteria were selected by ampicillin (100 μg/ml). The expression and purification of His-tagged protein were performed as previously described (Soong et al., 2013). Lipopolysaccharide (LPS) was removed by the Endotoxin Removal Kit (GenScript), which can effective reduce LPS levels to less 0.1 EU/ml.

### Vaccination of mice

Female C57BL/6J mice (6–8 weeks old) received subcutaneous (SC) injection of DnaK protein (100 μg) or intranasal (IN) injection of DnaK protein (20 μg) following anesthesia with ketamine/xylazine intraperitoneal injection. Another dosage of vaccination was given 2 weeks after the first vaccination. All procedures were performed according to protocols approved by the Johns Hopkins University Institutional Animal Care and Use Committee. Two weeks after the last vaccination, the mice were euthanized. Peripheral blood was collected by cardiac puncture and splenocytes were collected by mashing the spleens through a 100-μm cell strainer and following lysis of red blood cells by ACK lysing buffer (Quality Biological). For BCG vaccination, *Mycobacterium bovis* Karlson and Lessel (BCG Pasteur, ATCC) was grown in Middlebrook 7H9 broth (BD Difco) supplemented with 10% oleic acid-albumin-dextrose-catalase (OADC) (BD), 0.1% glycerol, and 0.05% Tween- 80 at 37°C on a shaker. BCG (10^6^ bacilli) at mid-logarithmic phase in 100 μl PBS was subcutaneously injected into C57BL/6J mice (6–8 weeks old) prior to aerosol challenge with Mtb.

### *In vivo* CD4^+^ T-cell depletion

For CD4^+^ depletion studies, 300 μg of the anti-CD4 monoclonal antibody, GK1.5 (BioXcell), was injected intraperitoneally into mice once and then 100 μg of antibody was injected 1 week later. To test whether intranasal DnaK vaccination could generate intrapulmonary antigen-specific T cells following peripheral CD4^+^ T-cell depletion, 200 μg of GK1.5 antibody was injected 1 day before the first vaccinationand then 100 μg of antibody was injected once weekly for 3 weeks.

### Enzyme-linked immunosorbent assay (ELISA)

Antigen-specific antibody responses were measured by ELISA, as described previously (Cheng et al., 2001), with minor modifications in coating and serum incubation. The 96-well microplate was coated with purified DnaK protein (1 μg/ml) overnight. After blocking, sera from vaccinated mice were diluted 1:100 with PBS, added to wells, and incubated at room temperature for 2 h.

### Intracellular cytokine staining and flow cytometry analysis

To detect antigen-specific CD4^+^ T-cell responses by IFN-γ and IL-17 intracellular staining, splenocytes were stimulated individually with recombinant DnaK (10 μg/ml) for 24 h at 37°C before addition of GolgiPlug (BD Pharmingen, San Diego, CA) overnight. After incubation, the splenocytes were washed once with FACScan buffer and then stained with PE-conjugated monoclonal rat anti-mouse CD4 (Clone RM4-5, BD Pharmingen, San Diego, CA). Cells were permeabilized using the Cytofix/Cytoperm kit (BD Pharmingen, San Diego, CA). Intracellular IFN-γ was stained with FITC-conjugated rat anti-mouse IFN-γ (Clone XMG1.2, BD Pharmingen, San Diego, CA) and IL-17 was stained with APC-conjugated rat anti-mouse IL-17 (Clone TC11-18H10.1, BioLegend). At necropsy, the lungs were perfused with 1 ml normal saline by direct injection into the right ventricle of the heart. A section of the lung was used for cytometry analysis and the tissue samples were incubated in 37°C for 1 h with intermittent agitation in RPMI medium (Gibco) containing collagenase D (1 mg/ml, Sigma), DNase (0.25 mg/ml, Sigma) and hyaluronidase type V (1 mg/ml, Sigma). The cells were then filtered through a 70-μm nylon filter mesh to remove undigested tissue fragments and washed with completed RPMI medium. To measure antigen-specific IL-17- or IFN-γ-releasing pulmonary T cells, the cells from each group were stimulated with DnaK protein (10 μg/ml) and intracellular cytokine staining was performed as described above. For analysis of pulmonary T cell surface markers, the T cells from the lungs were collected as described above. Then, the cells were stained with FITC-conjugated rat anti-mouse CD69 (clone H1.2F3, eBioscience), PE-conjugated rat anti-mouse CD4 and PerCP-conjugated rat anti-mouse CD44 (clone IM7, eBioscience). Flow cytometry was performed with a FACSCalibur flow cytometer, and the results were analyzed with FlowJo software.

### Aerosol infection of mice with *M. tuberculosis*

Wild-type *M. tuberculosis* (Mtb) H37Rv was grown in supplemented Middlebrook 7H9 broth, as previous described (Chuang et al., 2015). The mice were aerosol-infected using a Glas-Col Inhalation Exposure System (Terre Haute, IN) calibrated to deliver ~100 bacilli of wild-type Mtb H37Rv in a biosafety level-3 animal facility (Chuang et al., 2013). The mice were euthanized 28 days after aerosol challenge and the lungs were homogenized and plated for colony-formin units (CFU) (Chuang et al., 2013) to evaluate the protective efficacy of the DnaK vaccination.

### Statistical analysis

Group means were compared by one-way analysis of variance analysis (ANOVA) with Tukey-Kramer *post-hoc* analysis, using MedCalc for Windows, version 16.2.1 (MedCalc Software, Ostend, Belgium). Data from at least three biological replicates were used to calculate means and standard error of the mean (SEM) for graphing purposes. To compare differences between experimental and control groups, statistical analysis employed the unpaired Student's *t*-test, and a *p* < 0.05 was considered statistically significant.

## Results

### Vaccination of mice with recombinant DnaK generates antigen-specific IFNγ-secreting T cells in the spleens

**Figure 1:**
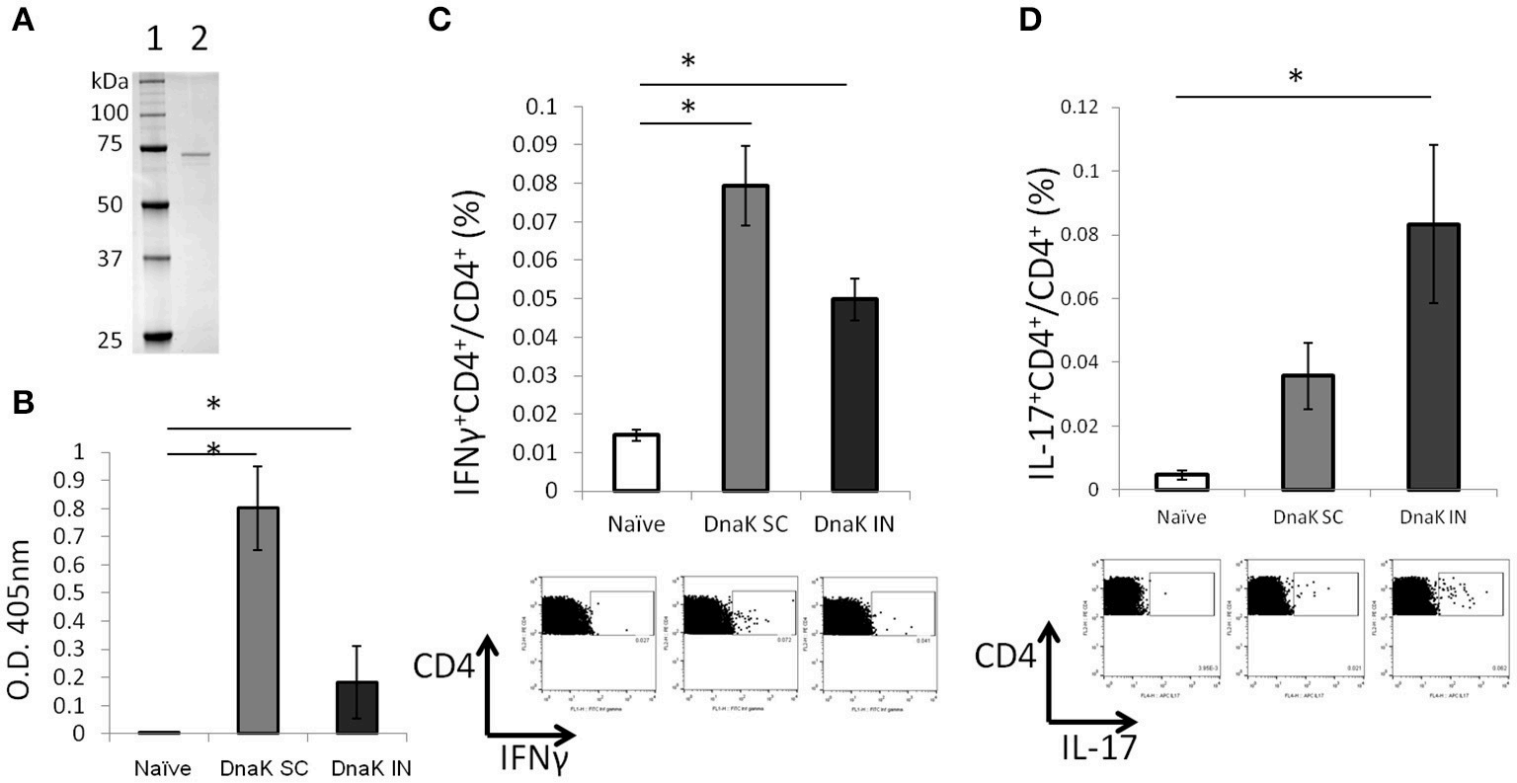
Vaccination with DnaK protein alone generates immunoglobulin responses and antigen-specific CD4^+^ T cells. **(A)** SDS/PAGE analysis of purified DnaK protein. Lane 1: protein ladder, Lane 2: Purified His-tag Dnak protein. C57BL/6J mice were vaccinated twice with DnaK (100 μg) by subcutaneous (SC) injection or with DnaK (20 μg) by intranasal (IN) injection. Immunological reponses were measured 2 weeks after the last vaccination. **(B)** Serum IgG responses to DnaK were measured by ELISA. Mean ± SEM, *N* = 3–4, ^*^p < 0.05 compared to the other two groups. **(C,D)** Splenocytes were stimulated with DnaK protein (10 μg/ml) and intracellular cytokine staining was used to detect antigen-specific CD4^+^ T cells secreting IFNγ **(B)** and IL-17 **(C)**. Mean ± SEM, *N* = 3–4, ^*^p < 0.05 compared to the other group.

Immunological responses were measured 2 weeks after SC or IN vaccination of C57BL/6J mice with recombinant DnaK (Figure 1A). Both routes of vaccine administration generated detectable IgG responses by ELISA, although SC vaccination generated significantly higher levels of IgG response compared to IN vaccination (Figure 1B). Intracellular cytokine staining revealed that DnaK vaccination by the SC or IN routes generated a significant increase in IFNγ-secreting CD4^+^ T cells in the spleen after stimulation with recombinant protein but there was no significant difference between the SC and IN groups (Figure 1C). However, only IN vaccination generated a significant increase in antigen-specific IL-17-secreting CD4^+^ splenocytes compared to the naïve group (Figure 1D, *p* < 0.05). The vaccination study was repeated by using a different batch of recombinant DnaK protein and the results were similar (data not shown).

### Intranasal vaccination with DnaK generates antigen-specific IL-17-secreting CD4^+^ T cells in the lungs

**Figure 2:**
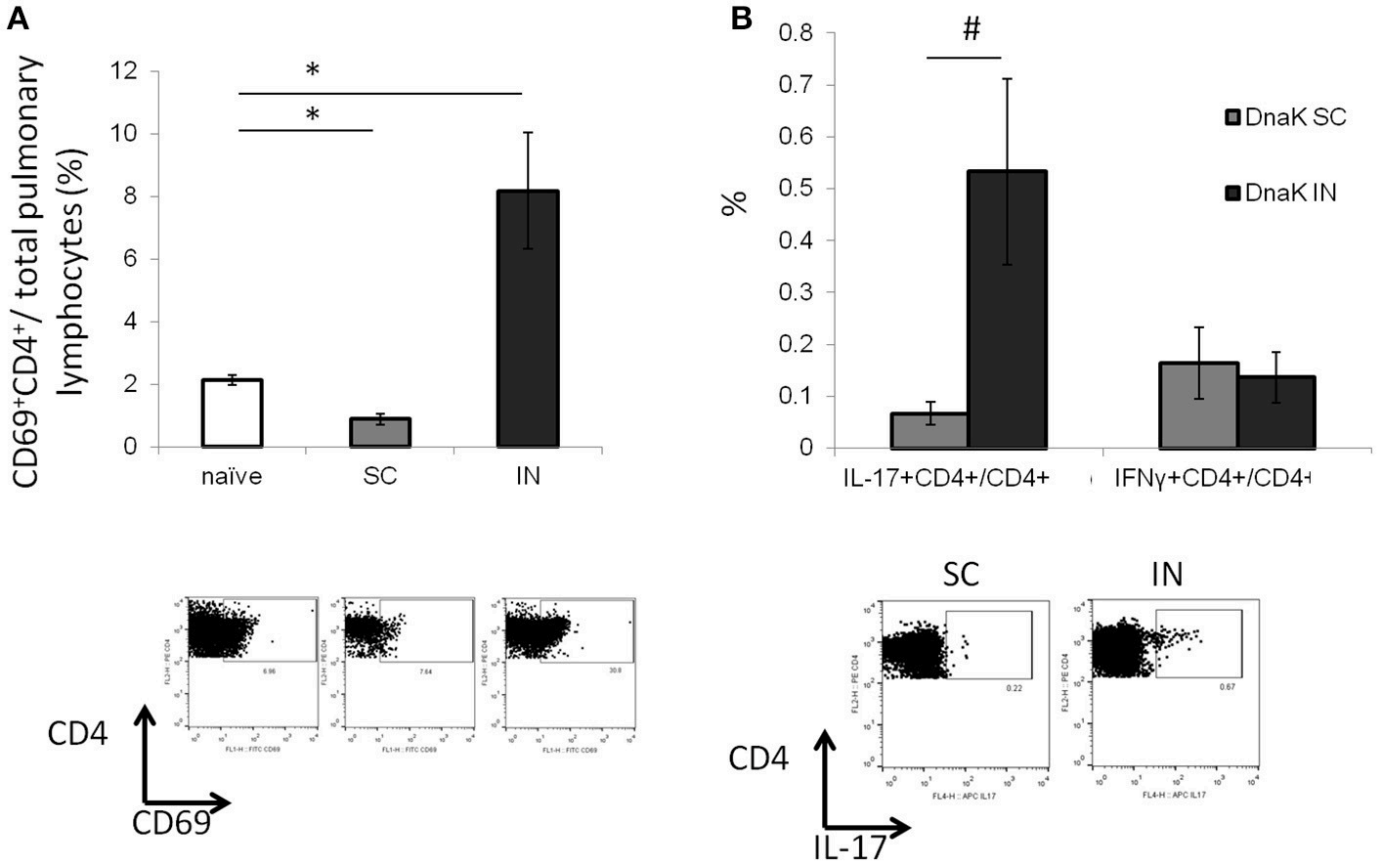
Intranasal vaccination with recombinant DnaK protein generates IL-17-secreting CD4^+^ T cells in the lungs. C57BL/6J mice were vaccinated twice with DnaK (100 μg) by subcutaneous injection or DnaK (20 μg) by intranasal injection. Parenchymal intrapulmonary T cells were analyzed 2 weeks after the last vaccine dose. **(A)** After tissue digestion, unstimulated intrapulmonary CD69^+^CD4^+^ T cells were measured by flow cytometry. *N* = 3–4, ^*^p < 0.05 compared to compared to the other two groups. **(B)** Intrapulmonary T cells were stimulated with recombinant DnaK (10 μg/ml) and antigen-specific CD4^+^ T cells were evaluated for IFNγ and IL-17 secretion by intracellular cytokine staining. Mean ± SEM, *N* = 3–4, #*p* < 0.05 compared to SC-vaccinated mice.

Lymphocytes from the lung parenchyma were harvested from DnaK-vaccinated mice and stimulated with recombinant DnaK protein (10 μg/ml) for 36 h. IN vaccination increased the proportion of CD69^+^CD4^+^ T cells in the lungs without antigen stimulation (Figure 2A, *p* < 0.05). Furthermore, intracellular cytokine staining revealed that IN vaccination generated significantly greater numbers of IL-17-secreting CD4^+^ T cells in the lungs compared to SC vaccination (Figure 2B, *p* < 0.05). Here we have shown that IN vaccination with DnaK generated mucosal-associated immunity.

### Intranasal vaccination with DnaK generates tissue-resident Th17 T cells in the lungs

**Figure 3:**
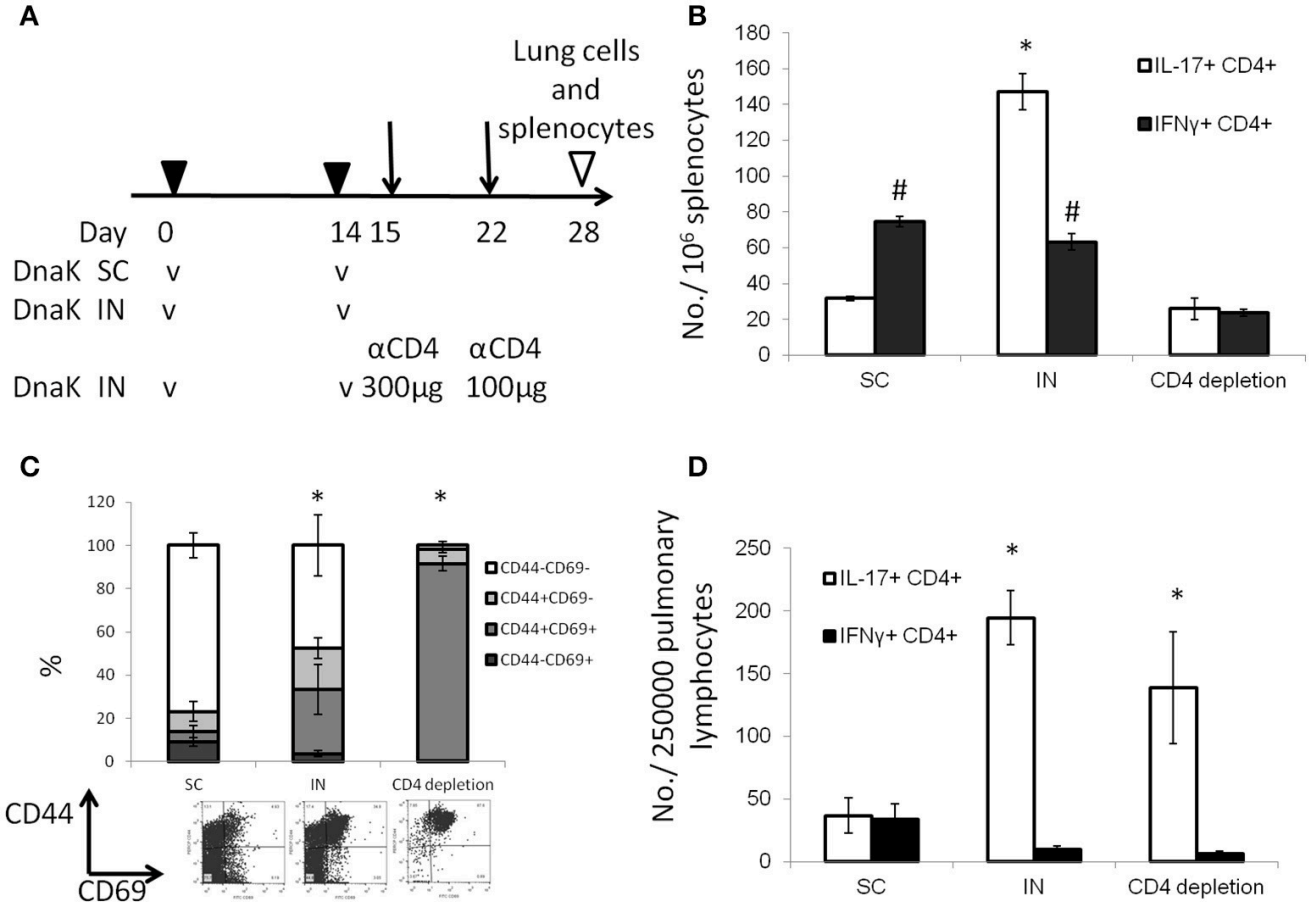
Intranasal vaccination with DnaK generates tissue-resident T cells. C57BL/6J mice were vaccinated twice two weeks apart with DnaK (100 μg) by subcutaneous injection or DnaK (20 μg) by intranasal injection. One group of intranasally vaccinated mice received intraperitoneal injection of anti-CD4^+^ neutralizing antibody (300 μg) 1 day after vaccination and another dose (100 μg) 1 week later. Parenchymal T cells were analyzed 2 weeks after the last vaccine dose. **(A)** Experiment scheme. **(B)** Splenocytes were stimulated with DnaK protein (10 μg/ml) and antigen-specific CD4^+^ T cells were assessed for IFNγ and IL-17 secretion by intracellular cytokine staining. Mean ± SEM, *N* = 3–4, ^*^p < 0.05 compared to the other two groups, #p < 0.05 compared to CD4^+^ depletion mice. **(C)** After tissue digestion, unstimulated intrapulmonary CD69^+^CD44^+^CD4^+^ T cells were measured by flow cytometry. Mean ± SEM, *N* = 3–4, ^*^p < 0.05 compared to the other two groups. **(D)** Intrapulmonary T cells were stimulated with recombinant DnaK (10 μg/ml) and antigen-specific CD4^+^ T cells were evaluated for IFNγ and IL-17 secretion by intracellular cytokine staining. Mean ± SEM, *N* = 3–4, ^*^p < 0.05 compared to compared to the other two groups.

To determine whether IN DnaK vaccination could generate lung tissue-resident T cells, mice vaccinated with recombinant DnaK by the IN route were treated with CD4^+^-neutralizing antibodies after booster vaccination (Figure 3A). CD4^+^ T-cell depletion was confirmed by measuring total CD4^+^ T-cell numbers by flow cytometry 1 week after treatment with anti-CD4^+^-neutralizing antibodies. There was 96.7 ± 3% reduction of CD4^+^ T cells in spleens following CD4^+^ T-cell depletion compared to the control group. Antigen-specific IL-17-secreting CD4^+^ T cells were significantly reduced in the spleen but not in the lungs following CD4^+^ T-cell depletion (Figures 3B,D). Without CD4^+^ T-cell depletion, IN DnaK vaccination significantly increased the number of CD44^+^CD69^+^CD4^+^ T cells (Figure 3C), which have been identified as lung tissue-resident T cells (Zens et al., 2016). Similarly, during systemic CD4^+^ T-cell depletion, there was a notable increase in the proportion of CD44^+^CD69^+^CD4^+^ T cells in the lungs of IN DnaK-vaccinated mice. Based these findings, IN vaccination with DnaK generates lung-resident IL-17-secreting antigen-specific CD4^+^ T cells, which were resistant to systemic CD4^+^ depletion.

### Intranasal vaccination with DnaK generates Th17 T cells in the lungs during CD4^+^ T-cell depletion

**Figure 4:**
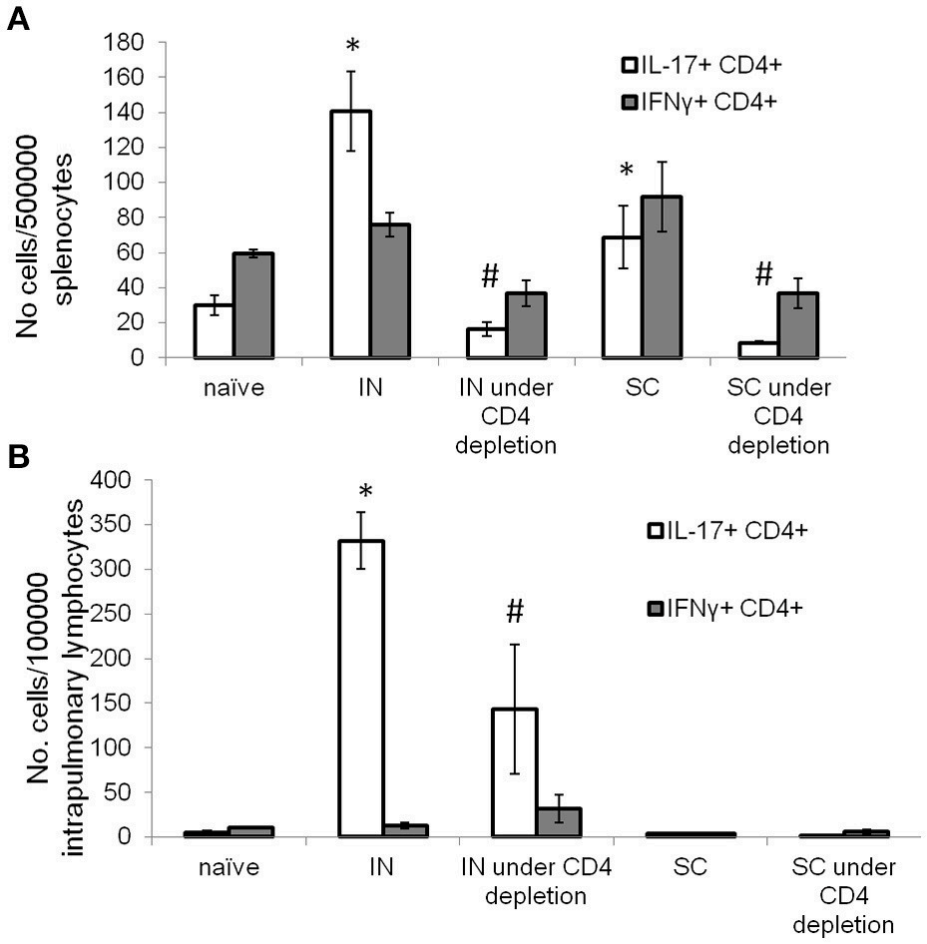
Intranasal DnaK vaccination generates antigen-specific IL-17^+^ CD4^+^ T cells in CD4-deficient mice. Control C57BL/6J mice were vaccinated with DnaK (100 μg) by subcutaneous injection or DnaK (20 μg) by intranasal injection then repeatly vaccinated again 2 week later. Separate group of mice were vaccinated as above, but also received anti-CD4^+^ neutralizing antibody 1 day before and during vaccination. Intrapulmonary T cells and splenocytes were analyzed 2 weeks after the last vaccine dose. Splenocytes **(A)** and intrapulmonary T cells **(B)** were stimulated with recombinant DnaK (10 μg/ml) and antigen-specific CD4^+^ T cells were evaluated for IFNγ and IL-17 secretion by intracellular cytokine staining. Mean ± SEM, *N* = 3–4, ^*^p < 0.05 compared to compared to the other two groups. #*p* < 0.05 compared to SC and IN groups.

During chronic HIV infection, CD4^+^ T cells counts are gradually reduced to <25% of the original numbers (Fauci et al., 1996). To determine if IN DnaK vaccination can generate immunity during CD4^+^ deficiency, control mice were vaccinated with DnaK (20 μg) by IN injection or DnaK (100 μg) by SC injection on Day 0 and Day 14. Experimental groups of mice received one of the vaccination schemes above, as well as intraperitoneal injection of anti-CD4^+^ neutralizing antibody one day before and during vaccination. Two weeks after the last vaccine dose, there was 65 ± 4% reduction of splenic CD4^+^ T cells in mice receiving anti-CD4^+^ neutralizing antibodies compared to those that did not receive neutralizing antibodies. Antigen-specific CD4^+^ T cells were measured in the spleens and lungs. Intracellular cytokine staining revealed a significant reduction in antigen-specific IL-17-secreting CD4^+^ splenocytes in the CD4^+^-depleted mice receiving IN or SC vaccination (Figure 4A). However, IN vaccination with DnaK still generated significant numbers of antigen-specific IL-17-secreting CD4^+^ cells in the lungs, even in CD4^+^-depleted mice (Figure 4B).

### DnaK vaccination offers protective immunity equivalent to BCG vaccination

**Figure 5:**
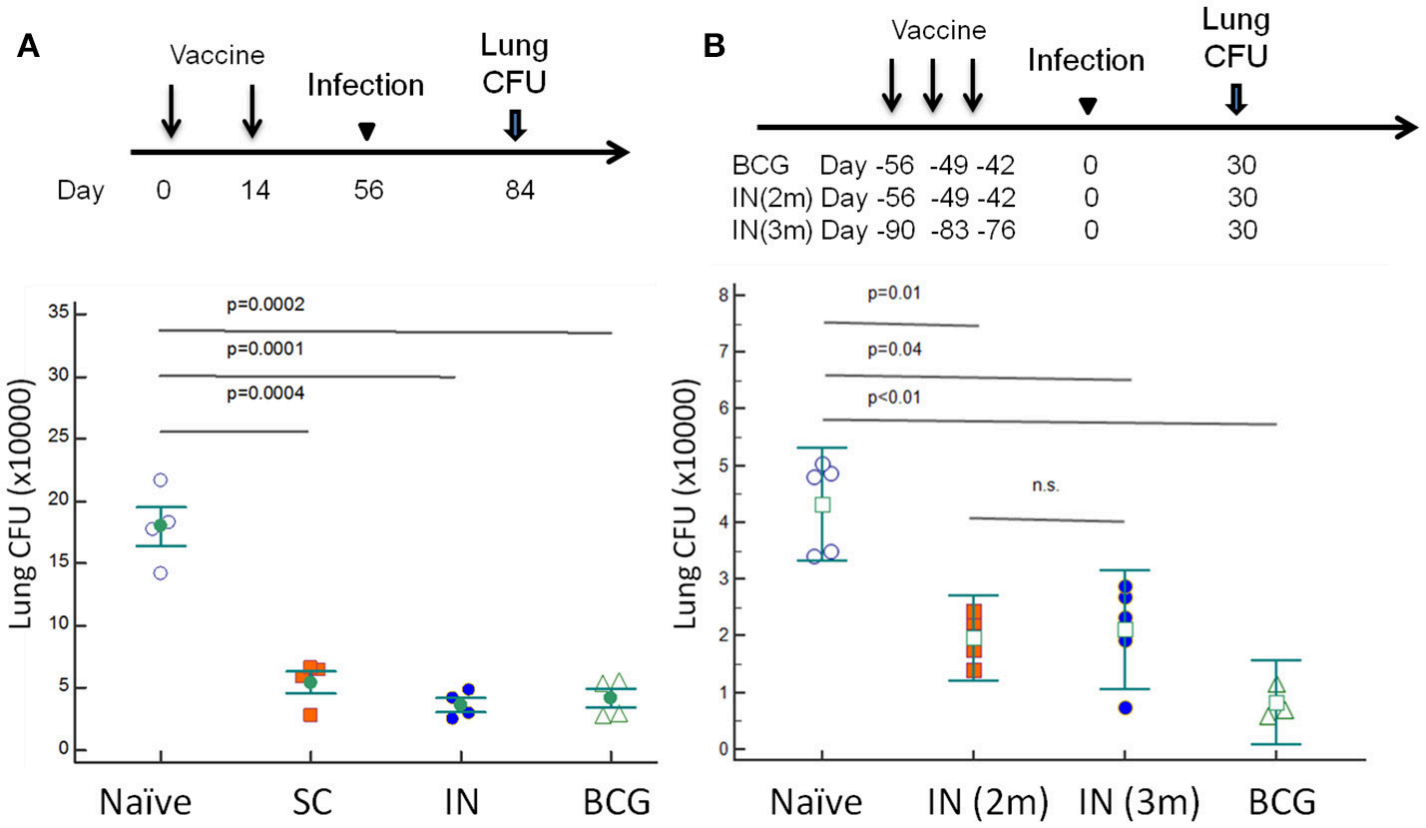
Vaccination with DnaK protein confers equivalent protective immunity as BCG vaccination. **(A)** C57BL/6J mice were vaccinated twice with DnaK (100 μg) by subcutaneous injection or DnaK (20 μg) by intranasal injection. Control mice were subcutaneously vaccinated with BCG Pasteur once. Eight weeks after vaccination, all mice were aerosol-challenged with virulent *M. tuberculosis* H37Rv. Four weeks after infection, the lung bacillary burden (log_10_ CFU) was determined. Mean ± SEM, ^*^p < 0.05 **(B)**. To confirm the protection of intranasal vaccination, C57BL/6J mice were vaccinated with DnaK (20 μg) intranasally once a week for a total of 3 weeks, at two (IN 2 m) or 3 months (IN 3 m) before H37Rv aerosal infection in another experiment. The vaccinated and unvaccinated group of mice were infected at the same time. Lung bacillary burden (log_10_ CFU) was determined 30 days after aerosol challenge. Mean ± SEM, ^*^p < 0.05.

C57BL/6J mice were vaccinated with DnaK (100 μg) by SC or IN injection. Another group of mice were vaccinated with 10^6^ BCG Pasteur via SC injection. Eight weeks after vaccination, the mice were challenged with wild-type Mtb H37Rv via the aerosol route. After 28 days of infection, the bacterial burden in the lungs was significantly lower in BCG- and DnaK-vaccinated groups (*p* > 0.05) compared to that in the unvaccinated group (Figure 5A) and there was no difference between BCG- and DnaK-vaccinated groups. To determine whether the protective effect of IN DnaK vaccination was dependent on the timing of vaccination relative to aerosol challenge and/or the specific DnaK protein preparation, C57BL/6J mice were vaccinated with an independently prepared Mtb recombinant DnaK protein via the IN route once a week for 3 weeks, either 90 days or 56 days before aerosol challenge (Figure 5B). Control mice were vaccinated via the SC route with 10^6^ BCG Pasteur strain, as described above, 56 days before aerosol challenge. All mice were aerosol-infected together and lung bacterial burden was assessed 30 days after infection. IN vaccination with DnaK provided consistent protection against Mtb aerosol challenge, including in the group that was vaccinated 3 months before challenge (*p* < 0.05 compared to unvaccinated group).

## Discussion

Previous studies have shown that DNA vaccination targeting mycobacterial DnaK (HSP70) protects mice against challenge with Mtb, although this protection was inferior to that afforded by BCG (Lowrie et al., 1997). Limited data are available directly comparing the protective efficacy of protein- and DNA-based TB vaccines and different vaccination routes may yield distinct immunological responses (Britton and Palendira, 2003). BCG vaccination by the IN route shows superior protective efficacy compared to the SC route (Aguilo et al., 2016; Perdomo et al., 2016), perhaps due to the induction of increased numbers of tissue-resident IL-17-secreting CD4^+^ T cells (Perdomo et al., 2016). Additional studies have also shown that vaccine presentation to the airways enhances control of Mtb infection in the lungs of mice (Derrick et al., 2014; Dai et al., 2016). In the current study, we show that IN vaccination with DnaK protein offers similar protection against Mtb challenge as SC BCG vaccination in mice, confirming the importance of the vaccine route of administration. This simple and effective vaccination strategy offers a new approach to protect against Mtb infection.

Following IN DnaK vaccination, the proportion of CD44^+^CD69^+^ CD4^+^ T cells was significantly expanded. In the CD4^+^ T-cell-deficient mice, the majority of intrapulmonary CD4^+^ T cells following IN vacination were CD44^+^CD69^+^ CD4^+^ T cells, which are a marker of tissue resident memory T cells (Zens et al., 2016). At the same time, there was a similar number of intrapulmonary IL-17^+^-secreting CD4^+^ T cells following CD4^+^ depletion. We showed that IN vaccination of DnaK alone can generate antigen-specific tissue-resident T cells in the lungs, which are maintained during systemic CD4^+^ T-cell depletion. Tissue-resident T cells offer protective immunity against herpes simplex virus 2 (Shin and Iwasaki, 2012; Iijima and Iwasaki, 2014), influenza (Zens et al., 2016), and Mtb (Perdomo et al., 2016). Mucosal vaccination with BCG generates lung resident T cells, which provide enhanced protection compared to SC BCG vaccination (Perdomo et al., 2016). Therefore, mucosal vaccination appears to be a promising strategy to protect against TB infection. Here we show that IN vaccination with the recombinant protein DnaK generates lung tissue-resident T cells and offers equivalent protective immunity as BCG against TB, with fewer theoretical risks associated with a live attenuated vaccine, especially in the immunocompromised host.

TLR2 is required for production of IL-17 by mucosal CD4^+^ T cells following IN vaccination and protection against different airway pathogens (Moffitt et al., 2014, 2015). In addition, TLR4 enhances IL-17 responses following mucosal vaccination with nanoemulsion adjuvants (Bielinska et al., 2010). DnaK can activate innate immunity through TLR2 and TLR4 (Bulut et al., 2005; Tsan and Gao, 2009), perhaps explaining its adjuvant properties during vaccination (Suzue and Young, 1996; Bulut et al., 2005), and offering a unique advantage as a TB preventive vaccine when administered via the IN route. In our study, we used the enodtoxin removal kit, which is effective in removing potential LPS contamination from purified DnaK protein. While we cannot entirely exclude the possibility of LPS-induced effects on T-cell responses following DnaK vaccination, we believe these effects are minimal for the following reasons. When using LPS as adjuvant during SC vaccination, there were significant CD4^+^ T cells with mixed secretion of IFNγ, IL-4, or IL-17 (Kim et al., 2009; O'donnell et al., 2014; Caucheteux et al., 2017). In contrast, in our study, IFNγ-secreting CD4^+^ T cells predominated in DnaK-vaccinated mice. In addition, the dose of LPS required to serve as adjuvant is greater than what could possibly remain after our protein purification. IN DnaK vaccination elicited antigen-specific IL-17^+^-secreting CD4^+^ T cells in the lungs, leading to similar protection as SC-administered BCG vaccination.

During HIV infection, there is significant reduction of Th17-secreting CD4^+^ T cells in the gastrointestinal tract, although Th17^+^ CD4^+^ T cells are relative preserved in broncheoalveolar lavage (Brenchley et al., 2008). Therefore, vaccination strategies capable of inducing Th17^+^ CD4^+^ T cells may potentially benefit HIV-infected individuals. Previous work has shown that administration of an Mtb Ag85A adenovirus vaccine to the respiratory mucosa generated Ag85A-specific CD8^+^ T cells in CD4^+^-depleted mice (Jeyanathan et al., 2010). Luminal CD8^+^ T cells can be sustained and renewed without peripheral T-cell recruitment (Jeyanathan et al., 2010). However, limited information exists regarding whether IN vaccination can generate mucosal antigen-specific CD4^+^ T cells when peripheral CD4^+^ T cells are depleted. Here we show that IN vaccination with recombinant DnaK generated significant numbers of tissue-resident IL-17^+^ CD4^+^ T cells in the lungs, even in mice unable to recruit systemic T cells following peripheral CD4^+^ T-cell depletion. This novel finding offers hope for novel TB vaccination strategies in patients with HIV/AIDS and other forms of cellular immune deficiency. It should be noted, however, that the presence of tissue-resident CD4^+^ T cells may reflect incomplete depletion due to reduced penetration of neutralizing antibodies into the lungs. Furthermore, it is unknown whether DnaK vaccine-induced lung-resident IL-17^+^ CD4^+^ T cells could persist during long-term CD4 deficiency, as in the setting of untreated AIDS. Further studies are needed to address these questions.

In summary, IN vaccination of mice with DnaK generated lung tissue-resident IL-17-secreting CD4^+^ T cells, offering similar protective efficacy against TB as SC BCG vaccination. The generation of intrapulmonary IL-17-secreting CD4^+^ T cells following IN DnaK vaccination was observed even following depletion of circulating CD4^+^ T cells, offering a novel potential vaccination strategy in immunocompromised hosts. Further studies are required to confirm the protective efficacy in other animal models, including non-human primates, and in the context of other immune deficiencies.

## Author contributions

Y-MC, PCK, and C-FH designed the experiments. Y-MC and MP performed experiments. Y-MC and C-FH performed analyses. PCK provided funding for the experiments. Y-MC, PCK, and C-FH wrote the manuscript.

### Conflict of interest statement

The authors declare that the research was conducted in the absence of any commercial or financial relationships that could be construed as a potential conflict of interest.
